# Factors Associated with Hospital Admission Decisions in Hematologic Emergency Department Patients: A Hierarchical Modeling Study

**DOI:** 10.3390/medicina62071385

**Published:** 2026-07-17

**Authors:** Osman Kuncan, Mustafa Burak Sayhan, Ömer Salt, Eray Çeliktürk

**Affiliations:** Department of Emergency Medicine, Faculty of Medicine, Trakya University, 22030 Edirne, Turkey

**Keywords:** emergency service, hospital, hematologic diseases, patient admission, triage, vital signs, mental status, logistic models, risk assessment

## Abstract

*Background and Objectives:* To identify factors associated with hospital admission decisions among hematologic patients presenting to the emergency department (ED) and to evaluate the incremental contribution of clinical, laboratory, and process-related variables using a hierarchical modeling framework. Materials and *Methods:* Single-center, retrospective observational cohort study conducted between January 2016 and December 2018. Adult patients for whom a hematology consultation was requested at ED presentation were included. Hierarchical block modeling was used as a sequential multivariable logistic regression approach in which clinical, laboratory, and process-related variables were entered in separate blocks. Process-related variables were defined as ED workflow-related factors, including mode of arrival, timing of presentation, and transfusion requirement. Model performance was assessed using the area under the receiver operating characteristic curve (AUC), Hosmer–Lemeshow goodness-of-fit test, calibration plot, calibration slope/intercept, and nested bootstrap internal validation. *Results:* Of 428 patients included, 297 (69.4%) were hospitalized. In the updated final model, impaired mental status (aOR 2.682; 95% CI 1.393–5.161), ambulance arrival (aOR 2.607; 95% CI 1.532–4.434), body temperature (aOR 1.399 per °C; 95% CI 1.049–1.866), respiratory rate (aOR 1.105 per breath/min; 95% CI 1.019–1.197), and heart rate (aOR 1.023 per bpm; 95% CI 1.007–1.040) were independently associated with hospital admission. Transfusion requirement showed an inverse association with admission (aOR 0.397; 95% CI 0.244–0.644), whereas platelet count showed the same direction of association but did not reach statistical significance in the updated final model. The final model demonstrated moderate apparent discrimination (AUC 0.771; 95% CI 0.725–0.818), with no statistically significant lack of fit on the Hosmer–Lemeshow test (*p* = 0.188). After nested bootstrap internal validation, the optimism-corrected AUC was 0.729. *Conclusions:* In hematologic patients presenting to the ED, admission decisions were associated mainly with acute physiological instability—particularly impaired mental status and abnormal vital signs—rather than by age or sex. These findings should be interpreted as exploratory associations with real-world disposition decisions, not as a validated tool for predicting objective outcomes such as mortality, ICU transfer, or 30-day adverse events.

## 1. Introduction

Hematologic disorders encompass a broad spectrum of conditions—ranging from hematologic malignancies to benign cytopenias—that share a common propensity for acute clinical deterioration. Infections, cytopenias, bleeding episodes, metabolic disturbances, and treatment-related toxicities may each precipitate life-threatening emergencies, often with overlapping presentations that complicate risk assessment and disposition decisions in the emergency department (ED) [1,2,3]. As a result, hematologic patients represent one of the most clinically heterogeneous and resource-intensive populations encountered in emergency care, with reported ED-to-admission rates consistently exceeding 50% in tertiary care settings [1,4].

Accurate and timely disposition decisions are therefore critical in this population. Delayed recognition of high-risk patients may result in preventable clinical deterioration, whereas unnecessary hospitalization contributes to increased healthcare utilization, prolonged ED crowding, and patient burden [5]. Accordingly, emergency department disposition decisions in hematologic patients represent clinically meaningful workflow endpoints reflecting both perceived disease severity and anticipated resource needs. The present study was not intended to develop a definitive admission score or validated clinical decision rule, but rather to explore how bedside physiological variables, laboratory findings, and process-related factors jointly shape hospital admission decisions in routine emergency care. Despite these challenges, structured risk stratification approaches specifically designed for hematologic patients at ED presentation remain limited. Existing early warning systems, including the Modified Early Warning Score (MEWS) and National Early Warning Score (NEWS), were primarily developed for general medical populations and may demonstrate reduced applicability in hematologic patients, particularly in the setting of immunosuppression, treatment-related toxicity, and atypical physiological responses [6,7]. Moreover, hematologic patients remain relatively underrepresented in conventional emergency department prediction studies, despite having distinct clinical trajectories and complex admission dynamics. Consequently, extrapolation of generalized ED prediction models to hematologic populations may be inadequate.

Previous investigations have partially addressed this gap. Kaplan et al. developed and validated a clinical prediction model for hospitalization among cancer patients presenting to the ED, demonstrating that routinely available triage variables may provide moderate discriminative performance (C-statistic 0.71–0.75) [4]. Similarly, studies evaluating mode of arrival and clinical acuity indicators have shown that prehospital and workflow-related factors may independently influence ED outcomes and disposition decisions [8,9]. However, these studies have largely focused on oncologic cohorts and have not comprehensively evaluated the combined contribution of clinical, laboratory, and operational variables within an exclusively hematologic ED population.

To our knowledge, no previous study has simultaneously evaluated these domains using a hierarchical block modeling approach in hematologic patients presenting to the ED. We therefore conducted this study to (1) identify clinical, laboratory, and process-related factors associated with real-world hospital admission decisions among hematologic patients presenting to the ED and (2) evaluate the incremental contribution of these variable domains using a hierarchical modeling framework. We specifically aimed to determine whether early indicators of physiological instability outweigh age, sex, and isolated laboratory variables in emergency department disposition decisions among hematologic patients.

## 2. Materials and Methods

### 2.1. Study Design and Setting

This study was designed as a single-center, retrospective, observational cohort study and is reported in accordance with the Strengthening the Reporting of Observational Studies in Epidemiology (STROBE) guidelines [10]. All patients presenting to the emergency department between 1 January 2016 and 31 December 2018 for whom a hematology consultation was requested were evaluated. Data were obtained from the hospital information management system and electronic medical records. Ethical approval was obtained from the Institutional Review Board (protocol code: 2018/20, approved on 19 November 2018). The requirement for informed consent was waived because of the retrospective design of the study and the use of anonymized patient data.

### 2.2. Study Population

All patients aged 18 years and older who presented to the adult emergency department during the study period and for whom a hematology consultation was requested were eligible for inclusion. Hematologic patients were defined as individuals with either hematologic malignancies or benign hematologic disorders who required evaluation by the hematology service during the emergency department visit. Patients were identified through hematology consultation request records in the hospital information management system, rather than by ICD codes alone, to capture the real-world population evaluated at the hematology–emergency medicine interface.

The following exclusion criteria were applied: age < 18 years, repeated hematology consultations during the same ED visit, records not fulfilling the eligibility criteria, and missing essential clinical or laboratory data. Missing essential data were defined as absence of ≥1 vital sign or ≥2 laboratory parameters at presentation. Missing data were handled using complete-case analysis, and no imputation was performed. Analytic sample size was assessed during model construction for each hierarchical model, and all multivariable analyses were performed using complete-case data.

**Figure 1:**
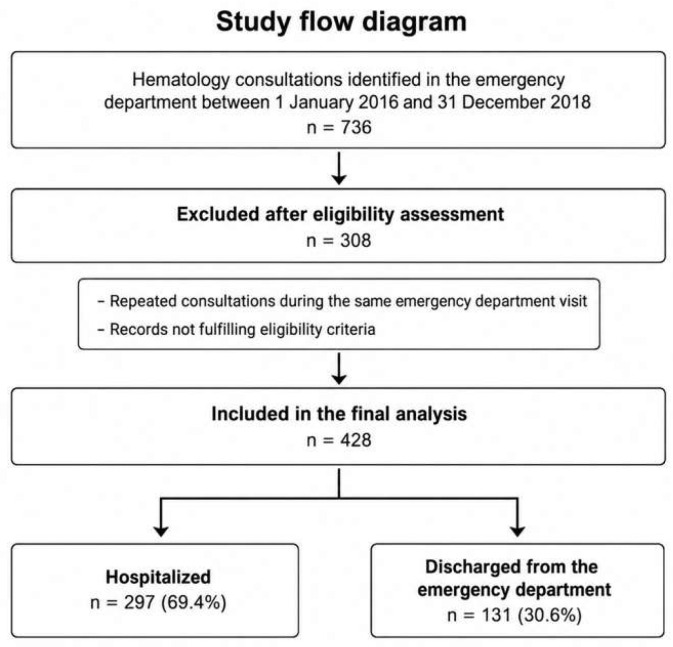
Study flow diagram.

During the study period, 736 hematology consultation records were screened; 308 were excluded after eligibility assessment, and 428 patients were included in the final analysis, as detailed in Figure 1. The screening log did not allow reliable separation of exclusions attributable specifically to missing essential data from exclusions due to repeated consultations or failure to meet eligibility criteria. Therefore, a missing-data-specific exclusion count could not be reported. However, all 428 included patients had complete data for the variables used in the hierarchical and final multivariable models, and the complete-case analytic sample remained unchanged across all models. Because the primary objective of the study was to evaluate real-world emergency department disposition decisions at the hematology consultation interface, patients with both malignant and benign hematologic disorders were analyzed within a unified emergency care framework despite underlying diagnostic heterogeneity.

### 2.3. Data Collection and Variables

Demographic, clinical, and laboratory data were systematically extracted from electronic medical records by trained data collectors using a standardized data extraction form. A random subset of records was independently reviewed by a second investigator to ensure consistency of data extraction. Variables were categorized into three main groups—clinical, laboratory, and process-related—according to their theoretical mechanism of influence on clinical decision-making and hospital admission, consistent with the hierarchical modeling framework described in the Introduction. The hierarchical block structure and variable overview are summarized in Appendix A.

### 2.4. Clinical Variables

Indicators of physiological instability included vital parameters recorded at the time of triage: systolic blood pressure (mmHg), heart rate (beats per minute), respiratory rate (breaths per minute), body temperature (°C), and mental status assessed using the AVPU (Alert–Voice–Pain–Unresponsive) scale. Impaired mental status was defined as any AVPU response other than “Alert” (AVPU ≠ A). Age (years) and sex were included as demographic variables within the clinical variable block. Comorbidity burden was recorded but not formally scored due to the heterogeneity of underlying diagnoses in the study population. Ethnicity was not routinely recorded in the emergency department electronic records and was therefore not included as an analyzable demographic variable.

### 2.5. Laboratory Variables

Markers of biological impairment included laboratory parameters obtained at initial ED presentation: hemoglobin level (g/dL), platelet count (×10^3^/µL), white blood cell count (×10^3^/µL) or absolute neutrophil count (ANC, ×10^3^/µL) when available, and serum creatinine level (mg/dL). Electrolyte parameters (sodium, potassium, chloride), serum glucose, and urea were also collected and included in the univariable screening stage. All laboratory values were obtained from samples drawn at triage or within the first hour of ED presentation.

### 2.6. Process-Related Variables

Process-related variables were defined as factors reflecting the operational and workflow characteristics of emergency department care rather than direct indicators of underlying disease severity. These variables capture how patients enter and are managed within the emergency care system and were included to reflect the multidimensional and partly institutional nature of admission decisions, as outlined in the Introduction.

Accordingly, process variables included: mode of arrival (ambulance vs. walk-in), timing of presentation (working hours defined as weekdays between 08:00 and 17:00; off-hours defined as nights, weekends, and public holidays), transfusion requirement during the ED visit (yes vs. no), and number of additional non-hematology consultations requested (0/1/≥2).

### 2.7. Outcomes

The primary outcome was hospital admission (vs. discharge from the ED). Admission was defined as any inpatient hospitalization following the ED visit, regardless of ward destination (general ward, hematology unit, or intensive care unit). Patients discharged after short-term ED observation without inpatient ward transfer were classified as discharged. This outcome was considered a real-world disposition endpoint reflecting clinician decision-making and anticipated monitoring needs, rather than an objective adverse clinical endpoint such as mortality, ICU transfer, or 30-day events.

### 2.8. Statistical Analysis

To address the study objective of evaluating the incremental contribution of each variable domain to model performance, hierarchical block modeling was applied as a sequential multivariable logistic regression strategy, in which theoretically defined blocks of variables are entered into the model in a prespecified order [11]. In this approach, variables were entered sequentially into three predefined blocks based on their clinical category: Block 1 (clinical variables), Block 2 (laboratory parameters), and Block 3 (process-related variables). Process-related variables referred to ED workflow-related factors rather than direct markers of underlying disease severity, including mode of arrival, timing of presentation, transfusion requirement, and additional non-hematology consultations. This approach enabled quantification of the incremental contribution of each domain to model performance while preserving a clinically interpretable structure that reflects real-world ED decision-making. This modeling strategy was selected a priori to reflect the sequential nature of real-world emergency department workflow, in which initial disposition decisions are generally initiated using immediately available bedside physiological assessment before laboratory interpretation, consultation processes, and operational factors become fully integrated into clinical decision-making. This framework was specifically designed to evaluate whether early physiological instability parameters remain the dominant determinants of emergency department disposition decisions after accounting for laboratory and operational factors.

Statistical analyses were performed using IBM SPSS Statistics for Windows, Version 20.0 (IBM Corp., Armonk, NY, USA). Continuous variables were assessed for normality using the Kolmogorov–Smirnov test and visual inspection of Q-Q plots and are presented as mean ± standard deviation (SD) or median (interquartile range [IQR]), as appropriate. Categorical variables are presented as number (n) and percentage (%). Group comparisons between hospitalized and discharged patients were performed using the independent samples *t*-test or Mann–Whitney U test for continuous variables and the chi-square test or Fisher’s exact test for categorical variables, as appropriate.

Univariable logistic regression analyses were initially performed within each predefined variable group to explore associations with hospital admission. Candidate variables for multivariable modeling were pre-specified based on clinical plausibility and prior literature, and univariable screening was then used to support model construction. Variables with *p* < 0.10 in univariable analysis, together with variables considered clinically important a priori, were considered for inclusion in the multivariable model. Thus, univariable associations informed but did not solely determine inclusion in the multivariable model.

To minimize the risk of overfitting, the number of predictors was considered relative to the number of outcome events using the events-per-variable (EPV) principle, targeting a minimum EPV ratio of 10. Given the retrospective design and consecutive patient inclusion over a predefined study period, a formal a priori sample size calculation was not performed. The refined final model included seven predictors and 297 admission events, yielding an events-per-variable ratio well above the conventional threshold of 10 and supporting the stability of the logistic regression estimates.

Multicollinearity among candidate variables was assessed using variance inflation factors (VIFs). The Modified Early Warning Score (MEWS) was not included in multivariable analyses because its component physiological variables were entered individually, resulting in conceptual and statistical redundancy. Variables were entered sequentially according to the predefined hierarchical block structure. The final model was derived from the full hierarchical model after removal of variables lacking both statistical significance and clinical justification for retention. Results are presented as adjusted odds ratios (aORs) with 95% confidence intervals (CIs). The final model was additionally re-estimated after excluding transfusion requirement, and this analysis was reported as a sensitivity analysis.

Model performance was evaluated in terms of discrimination, calibration, and internal validity. Discrimination was assessed using the area under the receiver operating characteristic (ROC) curve (AUC) with 95% confidence intervals. Calibration was assessed using the Hosmer–Lemeshow goodness-of-fit test, calibration plot, calibration slope, and calibration intercept. Model performance was compared across the three hierarchical blocks to quantify the incremental contribution of laboratory and process-related variables beyond clinical predictors alone. Internal validation was performed using 500 bootstrap resamples. In each bootstrap sample, the model-building process was repeated from the full hierarchical candidate model using backward variable selection, and optimism-corrected performance estimates were calculated. All tests were two-tailed, and a p-value < 0.05 was considered statistically significant.

## 3. Results

### 3.1. Baseline Characteristics

Of the 428 patients included in the final analysis, 297 (69.4%) were admitted to the hospital and 131 (30.6%) were discharged from the emergency department. The mean age of the study population was 60.5 ± 19.2 years. No statistically significant difference was observed between hospitalized and discharged patients with respect to age (60.5 ± 18.3 vs. 60.7 ± 21.0 years, respectively; *p* = 0.930). Similarly, sex distribution did not differ significantly between groups (male sex: 59.3% vs. 52.7%; *p* = 0.135). Ambulance arrival was significantly more frequent among hospitalized patients compared with discharged patients (40.7% vs. 21.4%; *p* < 0.001). In contrast, the frequency of off-hours presentation did not differ significantly between groups (51.2% vs. 44.3%; *p* = 0.303). Detailed baseline characteristics are presented in Table 1.

### 3.2. Univariable Logistic Regression Analysis

Univariable logistic regression analysis identified several clinically relevant signals associated with hospital admission (Table 2). The strongest clinical association was observed for impaired mental status (AVPU ≠ A; OR: 5.520; 95% CI: 2.74–11.10; *p* < 0.001). Abnormal vital signs, including higher respiratory rate, higher heart rate, and elevated body temperature, were also associated with admission. Among process-related variables, ambulance arrival was associated with increased odds of hospital admission, whereas transfusion requirement showed an inverse association. Platelet count demonstrated a borderline inverse association with hospital admission. Off-hours presentation, hemoglobin level, white blood cell count, serum urea, serum creatinine, and MEWS were not significantly associated with hospital admission in univariable analysis. Detailed univariable regression results are presented in Table 2.

### 3.3. Multivariable Logistic Regression Analysis

Multivariable logistic regression analysis was performed using the predefined hierarchical block modeling strategy. Three sequential block models were constructed. All hierarchical models were refitted using the same complete-case analytic sample (n = 428). A refined final model was then derived from Model 3 using post hoc removal of variables that lacked both statistical significance and a priori clinical justification for retention (Table 3). Therefore, the final model should be interpreted as a parsimonious exploratory model rather than as an additional prespecified hierarchical block. In the refined final model, four clinical variables, one laboratory variable, and two process-related variables were retained.

In the updated final multivariable model, impaired mental status remained independently associated with hospital admission (aOR: 2.682; 95% CI: 1.393–5.161; *p* = 0.003). Ambulance arrival was also independently associated with hospitalization (aOR: 2.607; 95% CI: 1.532–4.434; *p* < 0.001).

Among physiological parameters, body temperature (aOR: 1.399 per °C; 95% CI: 1.049–1.866; *p* = 0.022), respiratory rate (aOR: 1.105 per breath/min; 95% CI: 1.019–1.197; *p* = 0.015), and heart rate (aOR: 1.023 per bpm; 95% CI: 1.007–1.040; *p* = 0.004) remained independently associated with hospital admission. When interpreted across clinically meaningful ranges, these estimates corresponded approximately to an OR of 1.98 for a 30-bpm increase in heart rate and an OR of 1.65 for a 5 breaths/min increase in respiratory rate.

Among laboratory and process-related variables, transfusion requirement demonstrated an inverse association with hospitalization (aOR: 0.397; 95% CI: 0.244–0.644; *p* < 0.001). Platelet count showed the same direction of association but did not reach statistical significance in the updated final model (aOR: 0.999; 95% CI: 0.998–1.000; *p* = 0.089). Variables removed from Model 3 during derivation of the refined final model were age, sex, systolic blood pressure, hemoglobin, white blood cell count, sodium, potassium, chloride, glucose, urea, creatinine, off-hours presentation, and additional non-hematology consultations.

As a sensitivity analysis, the final multivariable model was re-estimated after excluding transfusion requirement. The main clinical associations remained stable: impaired mental status, ambulance arrival, body temperature, respiratory rate, and heart rate remained independently associated with hospital admission. Platelet count showed the same direction of association but did not reach statistical significance. The sensitivity model demonstrated moderate discrimination (AUC: 0.751), supporting that the principal clinical findings were not dependent on inclusion of transfusion requirement (Table 3).

**Figure 2:**
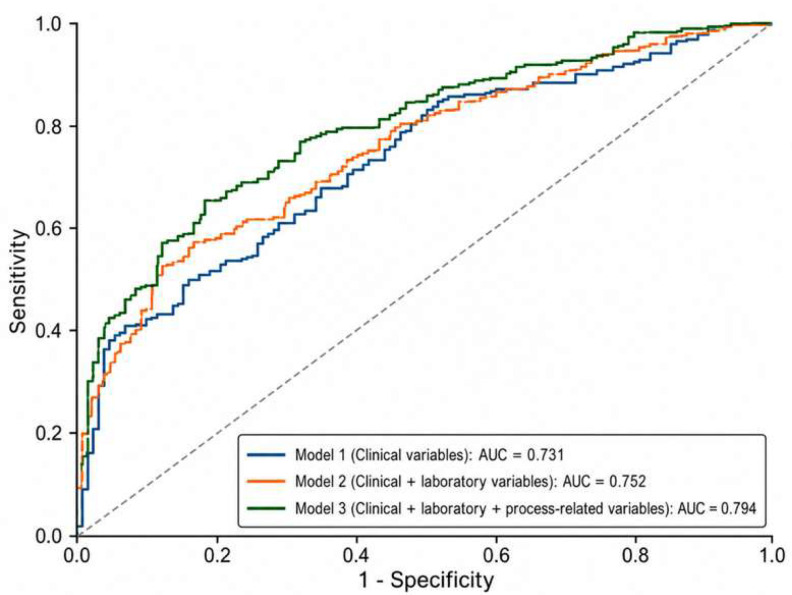
ROC curves illustrating the discriminative performance of Model 1 (clinical variables), Model 2 (clinical + laboratory variables), and Model 3 (clinical + laboratory + process-related variables) for hospital admission. The apparent AUC values were 0.731, 0.752, and 0.794, respectively. The dashed diagonal line represents the reference line of no discrimination, corresponding to an AUC of 0.50.

**Figure 3:**
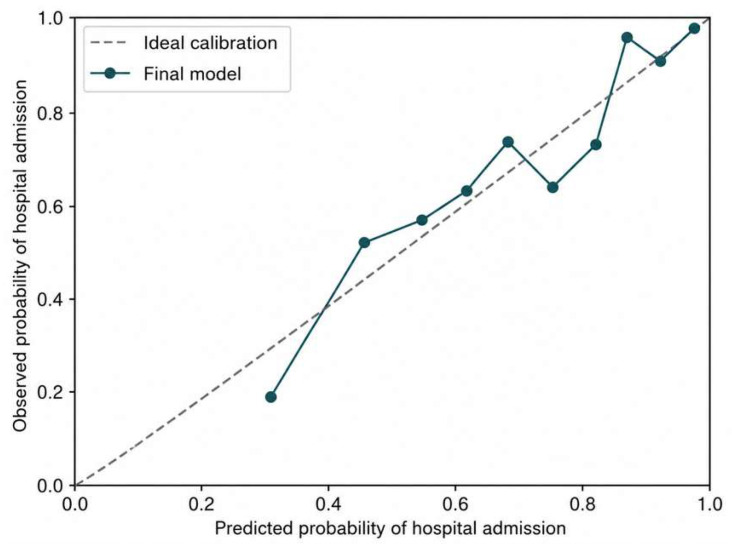
Calibration plot of the final multivariable logistic regression model. Calibration plot comparing predicted and observed probabilities of hospital admission across deciles of predicted risk. The apparent calibration slope was 1.000 and the apparent calibration intercept was 0.000. After nested bootstrap internal validation, the optimism-corrected calibration slope was 0.771 and the optimism-corrected calibration intercept was 0.004.

Model discrimination increased from Model 1 (clinical variables only; AUC: 0.731; 95% CI: 0.682–0.780) to Model 2 (clinical + laboratory variables; AUC: 0.752; 95% CI: 0.705–0.799), and was highest in Model 3 (clinical + laboratory + process-related variables; AUC: 0.794; 95% CI: 0.750–0.837). The refined final model demonstrated moderate apparent discrimination (AUC: 0.771; 95% CI: 0.725–0.818). The Hosmer–Lemeshow goodness-of-fit test showed no statistically significant lack of fit (χ^2^ = 11.25, df = 8, *p* = 0.188). Calibration was additionally assessed using a calibration plot, calibration slope, and calibration intercept. Nested bootstrap internal validation yielded an optimism-corrected AUC of 0.729, an optimism-corrected calibration slope of 0.771, and an optimism-corrected calibration intercept of 0.004. ROC curves for the hierarchical models are presented in Figure 2, comparative model performance and internal validation metrics are summarized in Table 4, and the calibration plot is shown in Figure 3.

## 4. Discussion

In this single-center retrospective cohort study, disposition decisions among hematologic patients presenting to the emergency department were driven predominantly by indicators of acute physiological instability, particularly impaired mental status and abnormal vital signs, rather than by age or sex. The refined final model demonstrated moderate apparent discrimination (AUC: 0.771). When all hierarchical models were refitted using the same complete-case analytic sample, the full hierarchical model showed the highest apparent AUC (0.794), whereas the refined final model was retained for parsimony and clinical interpretability rather than for maximal apparent discrimination.

The hospitalization rate observed in our cohort is consistent with—and in some reports exceeds—rates previously described in hematologic and oncologic emergency department populations. Among general cancer patients presenting to the ED, Caterino et al. reported a hospitalization rate of 57.2% in a large multicenter cohort, whereas studies restricted to hematologic malignancies have reported substantially higher admission frequencies [12]. Pettit et al., for example, reported hospitalization rates approaching 94% among patients presenting with febrile neutropenia [13]. The relatively high admission rate in the present study likely reflects the tertiary referral nature of the study center, inclusion of both malignant and benign hematologic disorders, and the use of hematology consultation requirement as an eligibility criterion—itself a marker of increased clinical complexity. Although advanced age is frequently associated with hospitalization in general ED populations, no significant age-related difference was observed between hospitalized and discharged patients in our cohort. Similarly, sex distribution did not differ significantly between groups. These findings suggest that, in hematologic patients, disposition decisions are driven predominantly by acute physiological and disease-related factors rather than demographic characteristics, consistent with prior hematologic emergency literature [1,3].

Arrival by ambulance was independently associated with a greater than 2.6-fold increase in the odds of hospitalization. Gefen et al. demonstrated that ambulance arrival independently predicted adverse outcomes among ED patients with chest pain, supporting mode of arrival as a surrogate marker of prehospital recognition of clinical severity [9]. In hematologic patients, conditions necessitating emergency transport—including febrile neutropenia, sepsis, acute bleeding, and altered consciousness—are inherently associated with higher admission likelihood [13,14]. The strong association identified in the present study suggests that clinical severity is frequently recognized before hospital arrival and that mode of arrival may serve as an immediately available marker of acuity during triage assessment.

Abnormalities in heart rate, respiratory rate, and body temperature were each independently associated with hospital admission. Among all clinical variables, impaired mental status demonstrated the largest effect size. This finding likely reflects severe underlying pathophysiological processes such as septic encephalopathy, metabolic derangement, or central nervous system involvement [6,15]. Importantly, although several physiological variables achieved statistical significance, their per-unit odds ratios were modest. For example, the aOR of 1.023 per 1 bpm increase in heart rate corresponds approximately to an OR of 1.98 for a 30 bpm increase and 2.48 for a 40 bpm increase, suggesting that heart rate is clinically meaningful mainly across larger differences rather than small per-bpm changes. Respiratory rate showed a larger per-unit association, with an approximate OR of 1.65 for a 5 breaths/min increase. These variables should therefore be interpreted cumulatively and in clinically meaningful ranges rather than through p-values alone. In contrast, altered mental status and ambulance arrival demonstrated more immediately interpretable effect sizes.

The association between thrombocytopenia and hospital admission reflects the unique biological characteristics of hematologic disease. Low platelet counts may indicate bone marrow failure, treatment-related myelosuppression, active disease progression, or increased bleeding risk, all of which may necessitate inpatient monitoring and intervention [3,16]. From a hematology perspective, platelet count may therefore carry particular practical weight because it can signal bleeding risk, marrow failure, or the need for disease-specific inpatient management. However, the per-unit effect size was clinically small: an aOR of 0.999 per 10^3^/µL increase in platelet count corresponds approximately to an OR of 0.90 for a 100 × 10^3^/µL increase. Therefore, platelet count should be interpreted as a complementary marker rather than as a dominant standalone determinant of admission.

The inverse association between transfusion requirement and hospital admission was counterintuitive and warrants cautious interpretation. The most plausible explanation is confounding by indication. Patients receiving transfusions in the ED may represent a clinically distinct subgroup, such as individuals with chronic stable anemia or predictable transfusion-dependent conditions, who can safely undergo treatment and subsequent discharge [15,16]. In addition, transfusion requirement arose during the ED visit and may therefore function as a process variable or mediator rather than a true baseline predictor, introducing potential temporal bias. Accordingly, this finding should not be interpreted as evidence that transfusion reduces hospitalization risk in acutely ill hematologic patients. Clinically, this pattern likely reflects a subgroup of relatively stable, transfusion-dependent patients in whom ED transfusion completed the immediate treatment need and allowed safe discharge, rather than any protective effect of transfusion in acutely unstable hematologic emergencies [1,15,16]. Consistent with this interpretation, the sensitivity analysis excluding transfusion requirement showed that the main associations for impaired mental status, ambulance arrival, and vital signs were retained. This supports that the principal clinical findings were not dependent on transfusion requirement. Nevertheless, because transfusion requirement arose during ED care, this association should be interpreted in the context of timing, patient selection, and confounding by indication rather than as a causal effect.

The hierarchical block modeling approach enabled structured evaluation of the relative contribution of clinical, laboratory, and process-related variables to disposition decisions [11]. Discriminative performance increased from the clinical-only model (AUC: 0.731) to the clinical + laboratory model (AUC: 0.752), corresponding to a modest incremental gain of 0.021 after addition of laboratory variables. A larger increase was observed after adding process-related variables in Model 3 (AUC: 0.794), corresponding to an additional gain of 0.042 beyond the clinical + laboratory model. This pattern suggests that laboratory parameters provided only limited additional discrimination, whereas process-related variables contributed a more noticeable incremental gain, mainly reflecting the influence of ambulance arrival and transfusion requirement. However, because process-related variables may partly reflect care pathways, patient selection, and institutional workflow rather than biological severity alone, this improvement should be interpreted cautiously. The refined final model was intentionally retained as a more parsimonious and clinically interpretable model and should not be interpreted as the model with the highest apparent AUC. Overall, bedside physiological assessment remained the core component of the model, while process-related variables added meaningful but context-dependent information.

The observed discriminative performance should be interpreted as moderate and exploratory. Kaplan et al. reported C-statistic values between 0.71 and 0.75 for hospitalization prediction among oncology patients presenting to the ED using triage-level variables [4]. For binary outcomes, the C-statistic is equivalent to the AUC and represents the probability that a model assigns a higher predicted probability to a hospitalized patient than to a non-hospitalized patient. Unlike previous ED prediction tools developed primarily for general cancer or oncology populations, the present study focused specifically on patients evaluated through the hematology consultation interface and simultaneously integrated clinical, laboratory, and process-related variables within a hierarchical framework. Although the apparent AUC of the refined final model was clinically informative, it does not indicate high-precision prediction. After nested bootstrap internal validation, the optimism-corrected AUC decreased from 0.771 to 0.729, indicating a degree of optimism in apparent model performance. These findings suggest that additional unmeasured factors, such as diagnosis-specific severity, treatment phase, bed availability, and physician-level decision-making, may contribute to admission practices. Therefore, the model should be interpreted as an exploratory description of variables associated with real-world disposition decisions rather than as a validated prediction tool intended to independently guide clinical decision-making.

It is also important to emphasize that the outcome analyzed in this study was actual hospital admission rather than an entirely objective measure of biological severity. Admission decisions may be partly shaped by institutional factors, including bed availability, local admission thresholds, hematology service pathways, and established organizational protocols [4,8,12]. Therefore, the model should be interpreted as describing associations with real-world ED disposition decisions, in which measurable clinical variables interact with clinician judgment and local practice patterns, rather than as a standalone measure of underlying disease severity [4,8,9]. Accordingly, the model should not be interpreted as a mortality, ICU admission, or long-term adverse outcome model; its relevance lies in characterizing real-world admission practices at the hematology–emergency medicine interface.

From a clinical implementation perspective, these findings may inform a simple bedside awareness checklist for hematologic patients presenting to the ED, rather than serving as a validated clinical decision rule. ED physicians and hematologists should pay particular attention to impaired mental status, tachycardia, tachypnea, fever, lower platelet count once initial laboratory results are available, and ambulance arrival [1,3,15,16]. The coexistence of these features should prompt closer monitoring and early hematology notification, but the decision to admit should continue to rely on clinician judgment, diagnosis-specific context, and local care pathways.

The next step should be prospective validation of these predictors in multicenter cohorts across different healthcare systems and admission pathways. If validated, the variables could be incorporated into electronic decision-support tools, ED triage protocols, or hematology consultation pathways to support structured risk stratification at the hematology–emergency medicine interface. Such implementation should be preceded by internal validation, external validation, calibration assessment, and subgroup-specific evaluation according to hematologic diagnosis and febrile status [11].

### Strengths and Limitations

One of the major strengths of this study is the inclusion of a relatively large consecutive cohort evaluated at the hematology–emergency medicine interface, together with simultaneous assessment of clinical, laboratory, and process-related variables within a unified hierarchical analytical framework. The hierarchical block modeling strategy enabled structured evaluation of the incremental contribution of each variable domain while preserving clinical interpretability.

Several limitations should nevertheless be acknowledged. The most important methodological limitation is the absence of diagnostic subclassification among hematologic disorders. Patients with acute leukemia, lymphoma, myeloma, myelodysplastic syndromes, and benign hematologic conditions were analyzed as a single heterogeneous population despite substantial differences in physiological presentation, treatment requirements, and admission thresholds across these entities. For example, patients with acute leukemia and those with chronic transfusion-dependent anemia may exhibit similar laboratory abnormalities despite fundamentally different clinical trajectories and hospitalization requirements. This heterogeneity represents an important source of residual confounding and limits disease-specific applicability of the model. Future studies should therefore stratify analyses according to hematologic diagnosis categories. Nevertheless, this heterogeneous structure also reflects the real-world composition of hematology consultation populations encountered in tertiary emergency departments, where disposition decisions are frequently made before definitive disease-specific stratification becomes fully established. Second, the retrospective single-center design limits external generalizability and introduces the possibility of documentation bias.

Third, the primary outcome of hospital admission represents a process-based disposition endpoint influenced by clinician judgment, institutional workflow, and resource availability rather than a fully objective clinical outcome such as mortality or ICU transfer. In addition, we could not directly assess clinician-level reasoning through structured interviews or compare admission thresholds across other specialty consultation populations; therefore, specialty-specific decision-making preferences could not be evaluated. Nevertheless, disposition decisions remain highly clinically relevant in emergency medicine because they directly reflect escalation-of-care decisions and anticipated monitoring requirements in real-world practice.

Although nested bootstrap internal validation was performed, no independent external validation cohort was available. Therefore, the generalizability of the model should be confirmed in external cohorts across different centers, patient populations, and admission pathways. Calibration was assessed using the Hosmer–Lemeshow test, calibration plot, calibration slope, and calibration intercept; however, these estimates were derived from the same retrospective cohort. Therefore, calibration performance should be reassessed in an independent validation cohort. In addition, because diagnostic subclassification was not available in a sufficiently structured form for robust subgroup modeling, we could not evaluate model performance separately in key subgroups such as hematologic malignancy versus benign hematologic disorders or febrile versus afebrile presentations.

Finally, although the model demonstrated moderate discrimination, the AUC below 0.80 likely reflects the inherently multifactorial nature of emergency department disposition decisions, which depend not only on measurable physiological variables but also on dynamic clinical judgment, consultation processes, and operational factors that cannot be fully captured within retrospective modeling. Future studies should include external validation, recalibration when necessary, and subgroup-specific performance assessment before routine clinical implementation.

## 5. Conclusions

Among hematologic patients presenting to the emergency department, real-world disposition decisions appeared to be associated primarily with indicators of acute physiological instability rather than by age, sex, or isolated laboratory abnormalities. Mental status, vital signs, and mode of arrival demonstrated the strongest and most clinically interpretable associations with hospitalization. Laboratory variables added only modest discrimination, whereas process-related variables provided a larger incremental contribution, likely reflecting the effects of ambulance arrival and transfusion requirement within real-world ED workflow. These findings support a physiology-centered approach to early emergency department assessment, while emphasizing that the present model remains exploratory, describes admission decisions rather than objective adverse outcomes, and requires prospective multicenter validation before clinical implementation.

## Figures and Tables

**Table 1:** Baseline Characteristics of Hematologic Patients Presenting to the Emergency Department According to Hospital Admission Status.

Variable	Total (n = 428)	Hospitalized (n = 297)	Discharged (n = 131)	*p*-Value
Age, years (mean ± SD)	60.5 ± 19.2	60.5 ± 18.3	60.7 ± 21.0	0.930
Male sex, n (%)	245 (57.2)	176 (59.3)	69 (52.7)	0.135
Female sex, n (%)	183 (42.8)	121 (40.7)	62 (47.3)	
Mode of arrival				
Walk-in	279 (65.2)	176 (59.3)	103 (78.6)	<0.001
Ambulance arrival	149 (34.8)	121 (40.7)	28 (21.4)	
Off-hours presentation	210 (49.0)	152 (51.2)	58 (44.3)	0.303

Data are presented as mean ± standard deviation (SD) or number (%), as appropriate. Continuous variables were compared using the independent samples *t*-test, and categorical variables were compared using the chi-square test. A *p*-value < 0.05 was considered statistically significant. Abbreviations: SD, standard deviation;

**Table 2:** Univariable Logistic Regression Analysis for Hospital Admission According to Predefined Variable Groups.

Variable	OR	95% CI	*p*-Value
Process-related: Ambulance arrival	1.995	1.23–3.21	0.004
Clinical: Impaired mental status (AVPU ≠ A)	5.520	2.74–11.10	<0.001
Process-related: Transfusion requirement (yes)	0.392	0.24–0.64	<0.001
Process-related: Off-hours presentation	1.240	0.81–1.89	0.303
Process-related: Non-hematology consultations (per additional consultation)	1.312	1.08–1.60	0.006
Clinical: Heart rate (per 1 bpm increase)	1.030	1.017–1.044	<0.001
Clinical: Respiratory rate (per 1/min increase)	1.194	1.117–1.275	<0.001
Clinical: Temperature (per 1 °C increase)	1.431	1.12–1.83	0.004
Clinical: Systolic blood pressure (per 1 mmHg increase)	0.999	0.991–1.009	0.981
Laboratory: WBC (×10^3^/µL)	1.009	0.998–1.020	0.104
Laboratory: Hemoglobin	0.974	0.912–1.041	0.441
Laboratory: Platelet count (per 10^3^/µL increase)	0.999	0.998–1.000	0.054
Laboratory: Sodium (mmol/L)	0.938	0.893–0.985	0.010
Laboratory: Potassium (mmol/L)	0.710	0.511–0.986	0.041
Laboratory: Chloride (mmol/L)	0.930	0.890–0.971	0.001
Laboratory: Glucose (mg/dL)	1.006	1.001–1.011	0.023
Laboratory: Urea (mg/dL)	1.006	0.999–1.013	0.119
Laboratory: Creatinine (mg/dL)	1.101	0.823–1.474	0.516
Clinical score: MEWS	1.012	0.884–1.158	0.863

Odds ratios (ORs) are presented per one-unit increase for continuous variables. Variables are labeled according to their predefined domain: clinical, laboratory, process-related, or clinical score. Reference categories are indicated in the table. A *p*-value < 0.05 was considered statistically significant. Abbreviations: OR, odds ratio; CI, confidence interval; WBC, white blood cell count;MEWS, Modified Early Warning Score; AVPU, Alert–Voice–Pain–Unresponsive; bpm, beats per minute;

**Table 3:** Final Multivariable Logistic Regression Model and Sensitivity Analysis Excluding Transfusion Requirement.

Variable	Final Model aOR (95% CI)	*p*-Value	Sensitivity Model aOR (95% CI)	*p*-Value
Clinical: Impaired mental status (AVPU ≠ A)	2.682 (1.393–5.161)	0.003	2.690 (1.411–5.127)	0.003
Process-related: Ambulance arrival	2.607 (1.532–4.434)	<0.001	2.464 (1.465–4.143)	0.001
Clinical: Temperature (per 1 °C increase)	1.399 (1.049–1.866)	0.022	1.366 (1.027–1.817)	0.032
Clinical: Respiratory rate (per breath/min increase)	1.105 (1.019–1.197)	0.015	1.099 (1.017–1.188)	0.016
Clinical: Heart rate (per bpm increase)	1.023 (1.007–1.040)	0.004	1.026 (1.010–1.042)	0.001
Process-related: Transfusion requirement	0.397 (0.244–0.644)	<0.001	Not included	—
Laboratory: Platelet count (per 10^3^/µL increase)	0.999 (0.998–1.000)	0.089	0.999 (0.998–1.000)	0.100
Apparent AUC	0.771 (0.725–0.818)	—	0.751	—

Adjusted odds ratios (aORs) with 95% confidence intervals (CIs) are presented. The refined final model was derived from the full hierarchical model after removal of variables that lacked both statistical significance and a priori clinical justification for retention. The sensitivity model was generated by re-estimating the final multivariable logistic regression model after exclusion of transfusion requirement. Variables removed from Model 3 during derivation of the refined final model were age, sex, systolic blood pressure, hemoglobin, white blood cell count, sodium, potassium, chloride, glucose, urea, creatinine, off-hours presentation, and additional non-hematology consultations. Platelet count was retained because of its clinical relevance in hematologic patients, although it did not reach conventional statistical significance in the updated final model. Abbreviations: aOR, adjusted odds ratio; CI, confidence interval; AUC, area under the receiver operating characteristic curve; AVPU, Alert–Voice–Pain–Unresponsive; bpm, beats per minute.

**Table 4:** Hierarchical Model Comparison, Discriminative Performance, and Internal Validation Metrics.

Performance Metric	Model 1	Model 2	Model 3	Final Model
Variable block	Clinical only	Clinical + Laboratory	Clinical + Laboratory + Process	Post hoc-refined model
Analytic sample, n	428	428	428	428
Admission events, n	297	297	297	297
Discharge events, n	131	131	131	131
Variables included	Age, sex, HR, RR, Temp, SBP, AVPU	+ Platelet, Hgb, WBC, Na, K, Cl, glucose, urea, creatinine	+ Ambulance arrival, off-hours presentation, transfusion requirement, consultations	AVPU, ambulance arrival, Temp, RR, HR, transfusion requirement, platelet count
No. of predictors	7	16	20	7
EPV	>10	>10	>10	>10
Apparent AUC (95% CI)	0.731 (0.682–0.780)	0.752 (0.705–0.799)	0.794 (0.750–0.837)	0.771 (0.725–0.818)
Hosmer–Lemeshow χ^2^, df, *p*-value	—	—	—	11.25, 8, 0.188
Mean bootstrap optimism for AUC	—	—	—	0.043
Bootstrap optimism-corrected AUC	—	—	—	0.729
Apparent calibration slope	—	—	—	1.000
Mean bootstrap optimism for calibration slope	—	—	—	0.229
Bootstrap optimism-corrected calibration slope	—	—	—	0.771
Apparent calibration intercept	—	—	—	0.000
Mean bootstrap optimism for calibration intercept	—	—	—	−0.004
Bootstrap optimism-corrected calibration intercept	—	—	—	0.004

All hierarchical models were refitted using the same complete-case analytic sample. The final model was derived post hoc from Model 3 for parsimony and clinical interpretability; therefore, its AUC should be interpreted as apparent model performance. Internal validation was performed for the refined final model using 500 bootstrap resamples. In each bootstrap sample, the model-building process was repeated from the full hierarchical candidate model using backward variable selection. Optimism was estimated as the difference between bootstrap-sample performance and performance when the bootstrap-derived model was applied to the original sample. Because platelet count was retained in the final model on clinical grounds despite not reaching conventional statistical significance, the bootstrap optimism correction should be interpreted as primarily reflecting the automated backward-selection component of model building. The 13 variables removed from Model 3 during derivation of the refined final model were age, sex, systolic blood pressure, hemoglobin, white blood cell count, sodium, potassium, chloride, glucose, urea, creatinine, off-hours presentation, and additional non-hematology consultations. Abbreviations: AUC, area under the receiver operating characteristic curve; CI, confidence interval; EPV, events-per-variable; HR, heart rate; RR, respiratory rate; Temp, body temperature; SBP, systolic blood pressure; AVPU, Alert–Voice–Pain–Unresponsive; Hgb, hemoglobin; WBC, white blood cell count; Na, sodium; K, potassium; Cl, chloride.

## Data Availability

The data presented in this study are available on reasonable request from the corresponding author.

## Appendix A. Hierarchical Block Modeling Structure and Variable Overview

**Table A1:** Variables Included in the Hierarchical Block Modeling Approach.

Block	Category	Variables
Block 1	Clinical variables	Age, sex, heart rate, respiratory rate, body temperature, systolic blood pressure, AVPU mental status
Block 2	Laboratory variables	Hemoglobin, platelet count, WBC/ANC, sodium, potassium, chloride, glucose, urea, creatinine
Block 3	Process-related variables	Mode of arrival, timing of presentation, transfusion requirement, additional consultations

**Table A2:** Summary of Variable Groups and Data Presentation.

Variable Group	Included Variables	Presented in Main Tables
Clinical	Age, sex, vital signs, AVPU	Table 1 and Table 2
Laboratory	Hemoglobin, platelet, WBC, electrolytes, creatinine	Table 2 and Table 3
Process-related	Arrival mode, timing, transfusion, consultations	Table 1 and Table 3

All variables included in the study are presented with their distributions or regression results in Table 1, Table 2 and Table 3 of the main manuscript, while hierarchical model structure and performance metrics are summarized in Table 4.
